# Olfactory perception of chemically diverse molecules

**DOI:** 10.1186/s12868-016-0287-2

**Published:** 2016-08-08

**Authors:** Andreas Keller, Leslie B. Vosshall

**Affiliations:** 1 Laboratory of Neurogenetics and Behavior, The Rockefeller University, 1230 York Avenue, Box 63, New York, NY 10065 USA; 2 Howard Hughes Medical Institute, New York, USA; 3 Kavli Neural Systems Institute, The Rockefeller University, New York, USA

**Keywords:** Olfaction, Psychophysics, Cheminformatics, Perceptual variability, Structure-odor-relationship, Odor descriptors, Perceptual familiarity

## Abstract

**Background:**

Understanding the relationship between a stimulus and how it is perceived reveals fundamental principles about the mechanisms of sensory perception. While this stimulus-percept problem is mostly understood for color vision and tone perception, it is not currently possible to predict how a given molecule smells. While there has been some progress in predicting the pleasantness and intensity of an odorant, perceptual data for a larger number of diverse molecules are needed to improve current predictions. Towards this goal, we tested the olfactory perception of 480 structurally and perceptually diverse molecules at two concentrations using a panel of 55 healthy human subjects.

**Results:**

For each stimulus, we collected data on perceived intensity, pleasantness, and familiarity. In addition, subjects were asked to apply 20 semantic odor quality descriptors to these stimuli, and were offered the option to describe the smell in their own words. Using this dataset, we replicated several previous correlations between molecular features of the stimulus and olfactory perception. The number of sulfur atoms in a molecule was correlated with the odor quality descriptors “garlic,” “fish,” and “decayed,” and large and structurally complex molecules were perceived to be more pleasant. We discovered a number of correlations in intensity perception between molecules. We show that familiarity had a strong effect on the ability of subjects to describe a smell. Many subjects used commercial products to describe familiar odorants, highlighting the role of prior experience in verbal reports of olfactory perception. Nonspecific descriptors like “chemical” were applied frequently to unfamiliar odorants, and unfamiliar odorants were generally rated as neither pleasant nor unpleasant.

**Conclusions:**

We present a very large psychophysical dataset and use this to correlate molecular features of a stimulus to olfactory percept. Our work reveals robust correlations between molecular features and perceptual qualities, and highlights the dominant role of familiarity and experience in assigning verbal descriptors to odorants.

**Electronic supplementary material:**

The online version of this article (doi:10.1186/s12868-016-0287-2) contains supplementary material, which is available to authorized users.

## Background

In olfaction, the conscious percept of a smell is often discussed in terms of perceived intensity, perceived pleasantness, and perceived olfactory quality (“garlicky,” “flowery,” etc.). The perceived intensity of a stimulus is the most basic and least ambiguous of these measures. Previous research has shown that only sufficiently heavy, volatile, and lipophilic molecules are odorous [1]. Molecular features such as molecular weight or the partial charge on the most negative atom correlate with perceived intensity. Several of these molecular features were used in regression equations that modeled the intensity of 58 molecules with impressive accuracy (R^2^ = 0.77−0.79) [2]. However this prediction has not been tested in an independent dataset, and a formal model that relates chemical structure to intensity has yet to be reported [3]. Several models have been developed to predict perceived pleasantness of an odorant based on its physical features [4–6]. Both molecular size [4, 6] and molecular complexity [5] correlate with perceived pleasantness. Molecular complexity is estimated from the variety of elements and structural features of the molecule [7]. There are also well-known predictions of olfactory quality. Molecules containing sulfur atoms are predicted to smell “garlicky,” whereas molecules containing ester groups are predicted to have a “fruity” smell. However these predictions of individual olfactory qualities have not yet been rigorously verified by testing a large number of subjects.

Two perceptual features complicate solving the stimulus-percept problem for olfaction. The first complication is that different individuals perceive the same molecules with different sets of functional odorant receptors [8–12]. These differences have been shown to influence perception [9, 12–16], and the same molecule is therefore often perceived differently by different individuals. This complication is not unique to olfaction. Colorblind individuals perceive the same visual stimulus differently from standard observers. However, in olfaction, the variability between different individuals is unusually large [17–19]. The second complication is that prior experience, cultural practices, motivational state, and non-olfactory information affect verbal reports of olfactory perception. The common co-occurrence of sweet tastes and odorants that are described as smelling “sweet,” for example, has led to the suggestion that odorants such as vanillin acquire their sweet smell quality by being experienced together with sweet tastes [20].

Furthermore, olfactory psychophysics suffers from a paucity of empirical data necessary to formulate theories to relate stimuli to percept. Many past attempts to solve the stimulus-percept problem for olfaction have used the same dataset published in 1985 by Andrew Dravnieks, who asked expert panelists to evaluate 138 different molecules using 146 standard semantic descriptors [21]. The purpose of the Dravnieks study was to develop a standard lexicon for describing olfactory stimuli of interest to the flavor and fragrance industry. Accordingly, both the molecules themselves and the semantic descriptors attached to them represent only a small number of possible odorants and percepts that humans can experience. Although there are alternative sources of data on the perceptual qualities of larger numbers of molecules, these are often based on the judgments of experts from companies that provide fragrance materials [22, 23]. Information from these sources is not standardized, and it can be difficult to assess how the data were obtained and how reliable they are. These constraints have slowed attempts to relate the molecular structure of an odorant to its conscious percept by human subjects.

To improve current predictions, perceptual data for a larger number of diverse molecules is needed. In this study, we present and analyze data on the perception of 480 structurally diverse molecules, many of which have not been tested before, at two concentrations. Another improvement of our dataset is that we provide individual responses in addition to the average perception of the group of subjects so that we do not mask individual perceptual variability. The motivation behind producing this dataset was to increase the number and diversity of molecules that can be used to test formal models that predict perceived smell based on features of the molecules. All raw data are being made freely available with the publication of this work to stimulate further analysis.

We found that intensity perception was strongly related to vapor pressure and molecular weight. We also uncovered correlations in intensity perception between certain pairs or clusters of stimuli whose intensity ratings varied between subjects. The presence of sulfur atoms biased molecules to be perceived as unpleasant. Conversely, pleasantness was correlated with molecular complexity. Finally, we discovered that familiarity strongly biases olfactory perception. Unfamiliar stimuli were less likely to receive a semantic descriptor and tended to be neither pleasant nor unpleasant. This suggests that semantic categorization of olfactory stimuli alone is unlikely to solve the stimulus-percept problem.

## Results

**Fig. 1:**
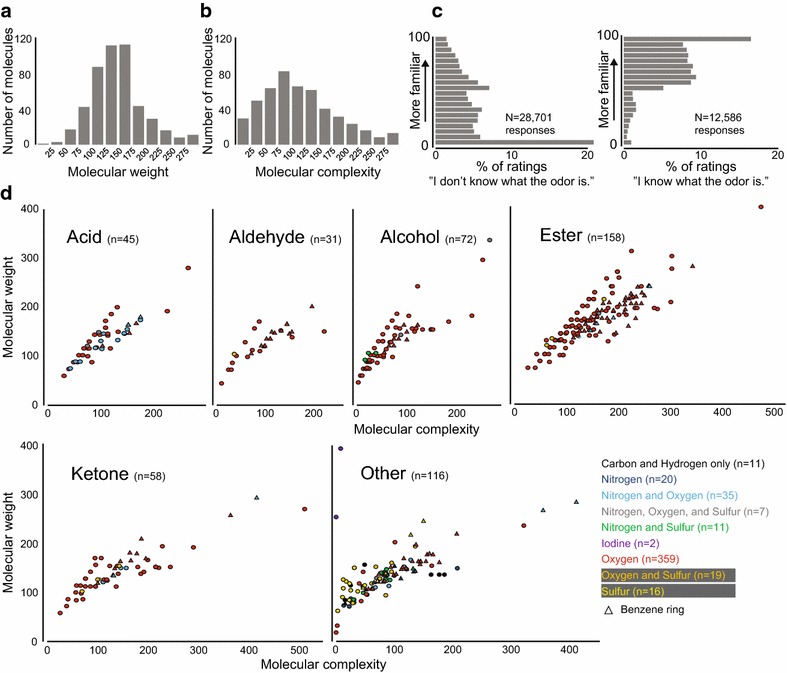
Molecules. **a**, **b **Molecular weight (**a**) and molecular complexity (**b**) of the molecules used in this study. **c** Histograms of familiarity ratings (0–100, binned in 20 units of 5) for stimuli that subjects identified as unknown (*left*) or known (*right*). *N* denotes the total number of responses across all stimuli and all subjects. **d** Molecular weight and molecular complexity parsed by chemical functionality

We tested the perception of 480 different molecules at two concentrations in 61 healthy subjects. 20 molecules were tested twice at both concentrations, for a total of 1000 stimuli. The molecules ranged in molecular weight from 18.02 (water) to 402.54 (tributyl-2-acetylcitrate) with a median of 144.24 (Fig. 1a), and in molecular complexity from 0 (water and iodine) to 514 (androstadienone) with a median of 109 (Fig. 1b). Many molecules had unfamiliar smells. Of the stimuli that subjects could perceive, 70 % were rated as unknown and were given low familiarity ratings (Fig. 1c, left), while those rated as known had high familiarity ratings (Fig. 1c, right). The molecules were structurally and chemically diverse, and some have never been used in prior psychophysical experiments. The 480 molecules had between 1 and 28 non-hydrogen atoms, and included 29 amines and 45 carboxylic acids. Two molecules contained halogen atoms, 53 had sulfur atoms, 73 had nitrogen atoms, and 420 had oxygen atoms (Fig. 1d).

**Fig. 2:**
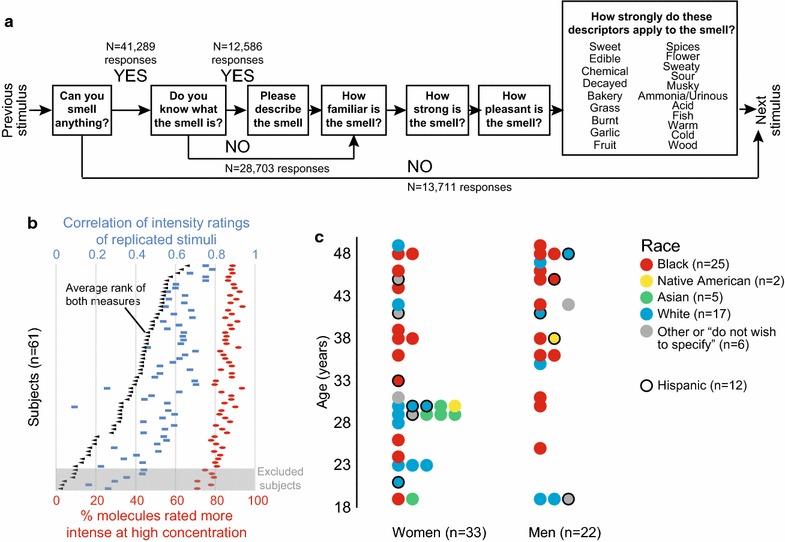
Subjects. **a** Sequence of prompts for each stimulus. *N* denotes the total number of responses across all stimuli and all subjects. **b** General olfactory performance of the 61 subjects who completed the study. Six subjects with the lowest rank in replicability of intensity ratings were excluded from further analysis. **c** Age, gender, and self-reported race and ethnicity of the 55 evaluated subjects

The 1000 stimuli were tested across 10 visits, and the order of visits and stimuli within visits were randomized. The sequence of prompts for each stimulus is shown in Fig. 2a. For questions about familiarity, intensity, pleasantness, and the 20 descriptors, subjects were presented with a slider that they moved along a line. The final position of the slider was translated into a scale from 0 to 100. The 20 descriptors were “edible,” “bakery,” “sweet,” “fruit,” “fish,” “garlic,” “spices,” “cold,” “sour,” “burnt,” “acid,” “warm,” “musky,” “sweaty,” “ammonia/urinous,” “decayed,” “wood,” “grass,” “flower,” and “chemical.”

**Fig. 3:**
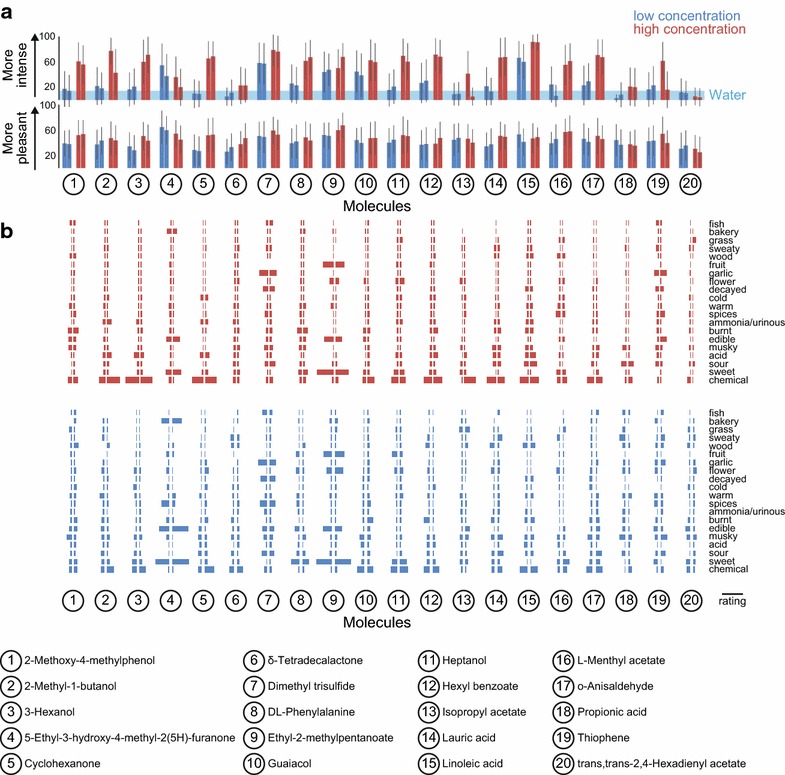
Repeated stimuli **a** Ratings for intensity (*top*) and pleasantness (*bottom*) for the 40 stimuli (20 molecules at two concentrations) each presented twice (mean ± S.D.). The intensity rating of water (14.44) is indicated by the *blue* shading. **b** Ratings of descriptors for high (*top*) and low (*bottom*) concentrations of 20 molecules each presented twice. Average ratings of descriptors for first (*left*-*facing bar plot*) and second (*right*-*facing bar plot*) presentations. Scale bar: rating of 50 on a scale of 0 to 100

Sixty one subjects completed all ten visits. We calculated and ranked the olfactory performance of all subjects by two measures that reflect the subjects’ overall olfactory acuity (for details see “Methods”), and excluded the six lowest-ranking subjects from further analysis (Fig. 2b). By convention in this and previous studies, we considered subjects in the bottom 10 % as possible malingerers or subjects suffering from low olfactory acuity. Data from the remaining 55 subjects formed the basis of all analysis in the paper (Fig. 2c) (Additional file 1). Twenty arbitrarily chosen molecules were presented twice at both concentrations throughout the study. In general, intensity and pleasantness (Fig. 3a) and descriptor usage (Fig. 3b) were consistent between the two presentations. For reasons that we do not understand, the intensity ratings and descriptors for high concentrations of 2-methyl-1-butanol (odorant 2), isopropyl acetate (odorant 13), and thiophene (odorant 19) differed substantially between the two presentations (Fig. 3a, b).

### Perception of the stimuli

**Fig. 4:**
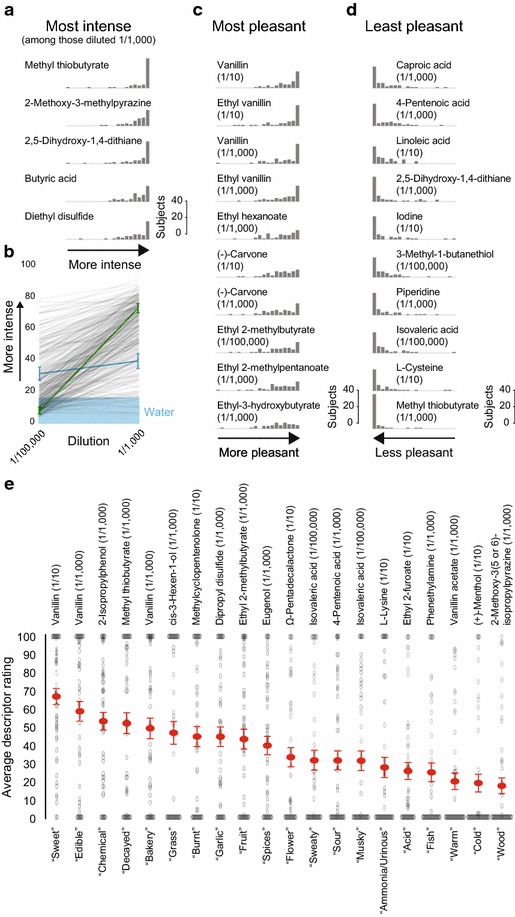
Perception of stimuli. **a** Histograms of intensity ratings for the five most intense of 437 stimuli presented at 1/1000 dilution (most intense on top). **b** Average intensity ratings of 437 molecules presented at 1/1000 and 1/100,000 dilutions, with standard error of the mean shown for two molecules [methyl salicylate (*green*) and methyl caprylate (*blue*)]. The intensity rating of water (14.44) is indicated by the *blue* shading. **c**, **d** The ten most pleasant (most pleasant on *top*) (**c**) and ten least pleasant of the 1000 stimuli (least pleasant on bottom) (**d**). **e** Descriptor rating of the stimuli most representative of each of the 20 descriptors. Individual ratings as well averages and standard errors (in *red*) are shown. Only the 778 stimuli perceived to be more intense than water (14.44) were included in this analysis. In **a**, **c**, **d** Histograms of subject ratings of intensity **a** or pleasantness **c**, **d** are plotted on a scale from 0–100, binned in 20 units of 5

437 of the 1000 stimuli were presented at the same dilution (1/1000). Among these, methyl thiobutyrate was rated to be most intense, followed by 2-methoxy-3-methylpyrazine, 2,5-dihydroxy-1,4-dithiane, butyric acid, and diethyl disulfide (Fig. 4a). On the opposite end of the intensity scale, 61 molecules when diluted 1/1000 were rated to be less intense than the average intensity rating of the two dilutions of water (14.44) (Additional file 1). In the majority of cases, stimuli presented at high concentration received higher intensity ratings than the same stimuli at low concentration (Fig. 4b).

In addition to perceived intensity, we tested perceived pleasantness. The two concentrations of vanillin and ethyl vanillin accounted for the four most pleasant stimuli in the study. Both concentrations of (−)-carvone and four different esters comprised the remainder of the ten most pleasant stimuli (Fig. 4c). The least pleasant stimulus was methyl thiobutyrate, which was also the most intense of the stimuli diluted 1/1000 (Fig. 4a). Another three of the ten least pleasant stimuli were also sulfur-containing molecules and four others were carboxylic acids (Fig. 4d).

Frequency of descriptor usage for a given molecule can reveal molecules that are representative of each of the odor quality descriptors (Fig. 4e). Methyl thiobutyrate (1/1000), the most intense and least pleasant stimulus, received the highest rating for “decayed.” Vanillin received the highest rating for “edible” (1/1000), “bakery” (1/1000), and “sweet” (1/10), and vanillin acetate (1/1000) was rated the “warmest” stimulus. Isovaleric acid (1/100,000) received the highest rating for both “musky” and “sweaty” (Fig. 4e).

### Variability in intensity perception

**Fig. 5:**
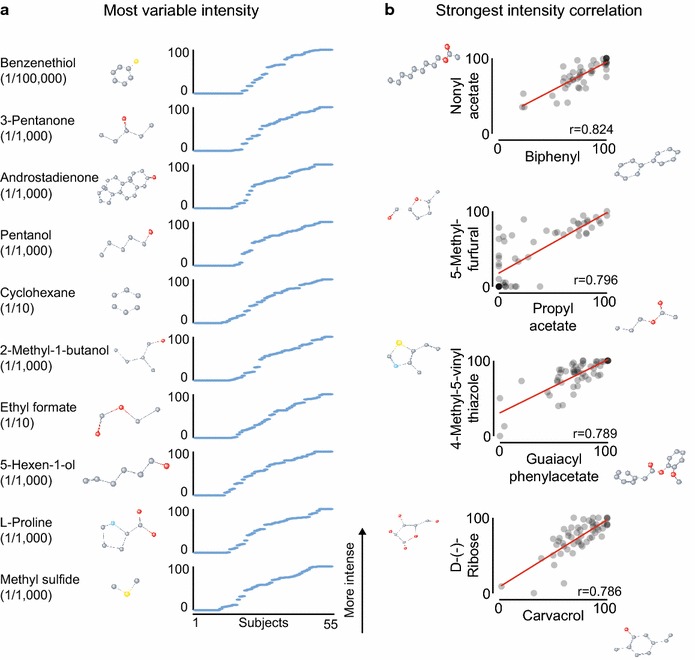
Variability in perception. **a** The ten stimuli with the most variability in intensity (most variable on *top*). **b** The four pairs of all stimuli with the largest correlation between intensity ratings (for each correlation: p < 1E−11). Only the 778 stimuli perceived on average to be more intense than water (14.44) were included in this analysis. In the chemical structures, atoms are colored as follows: carbon (*gray*), oxygen (*red*), nitrogen (*blue*), sulfur (*yellow*)

While some stimuli were rated by the majority of subjects to be between 85 and 100 on the intensity scale from 0 to 100 (Fig. 4a), and many others were perceived to be very weak by all subjects, a few stimuli showed great variability in intensity ratings (Fig. 5a). Androstadienone (1/1000), which is known to be perceived differently by different subjects [9, 15, 18], was the stimulus with the third most variable intensity perception after benzenethiol and 3-pentanone (Fig. 5a). The four stimulus pairs with the strongest correlation between perceived intensity ratings are shown in Fig. 5b.

### Descriptor usage and familiarity

**Fig. 6:**
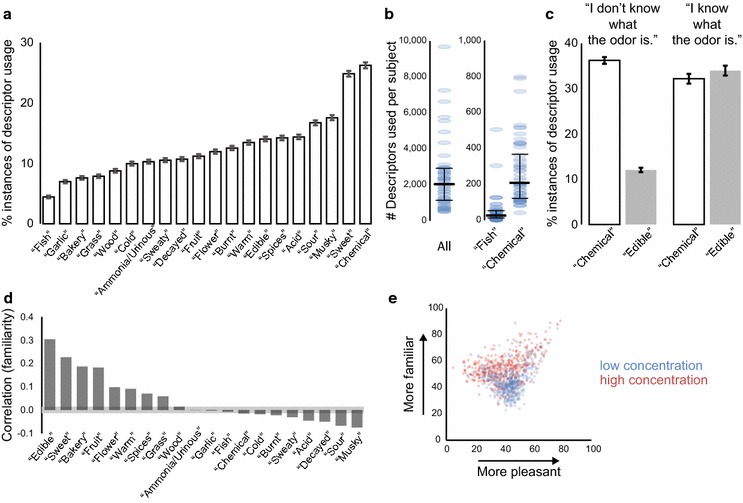
Descriptor usage and familiarity. **a** Descriptor usage for all subjects (with 99 % confidence interval indicated). 100 % would correspond to a descriptor assigned to all stimuli by all subjects. **b** Descriptor usage per subject. *Left* all descriptors (maximum possible value: 20,000). *Right* “chemical” and “fish” descriptors (maximum possible value: 1000). Data from 55 individual subjects (*blue*) and median and first and third quartiles (*black*). **c** Descriptor usage for “chemical” and “edible” for all stimuli (99 % confidence interval indicated), with responses divided according to unknown (*left*: N = 28,703 responses) and known (*right*: N = 12,586 responses) stimuli. 100 % would correspond to a descriptor assigned to all stimuli by all subjects. **d** Correlation between familiarity ratings and the ratings of 20 descriptors. The grey area along the x-axis indicates the range of correlations that are not statistically significant (after Bonferroni correction; N = 41,289, p > 0.0025). **e** Average familiarity and pleasantness ratings for 1000 stimuli

In addition to providing intensity and pleasantness ratings, subjects rated whether and how strongly each of 20 semantic descriptors applied to the stimuli. The most commonly used descriptor was “chemical,” and the least frequently used descriptor was “fish” (Fig. 6a). Subjects used very different strategies to apply descriptors to stimuli. One subject used only 508 descriptors for the 1000 stimuli, whereas another used 9678 descriptors (Fig. 6b, left). This is consistent with previous reports that descriptor usage is individualized [21]. The median number of total descriptors applied was 1900, meaning that the average subject applied about two descriptors to the average stimulus. Subjects also differed in how frequently they used individual descriptors (Fig. 6b, right). Three subjects did not apply the descriptor “fish” to any of the stimuli. At the other extreme, one subject applied this descriptor to more than half of the stimuli (505/1000). At the median, subjects applied “fish” to 21 stimuli. The two subjects who applied “fish” to more than 150 stimuli also applied the largest number of descriptors overall. The frequency of applying the descriptor “chemical” also varied between subjects. One subject applied it to only 80 stimuli, another to 798 stimuli.

Some descriptors were predominantly applied to familiar stimuli, whereas others were often used for unfamiliar molecules. For example, for unfamiliar odorants, “chemical” was a more common descriptor than “edible” (Fig. 6c, left), whereas both were used equally for familiar stimuli (Fig. 6c, right). Correlations between familiarity and the ratings for the 20 descriptors showed that “edible” was most strongly correlated with high familiarity (Fig. 6d). Unfamiliar stimuli tended to be neither pleasant nor unpleasant, whereas the most pleasant stimuli were also judged to be very familiar (Fig. 6e).

### Correlations between odor quality descriptors

**Fig. 7:**
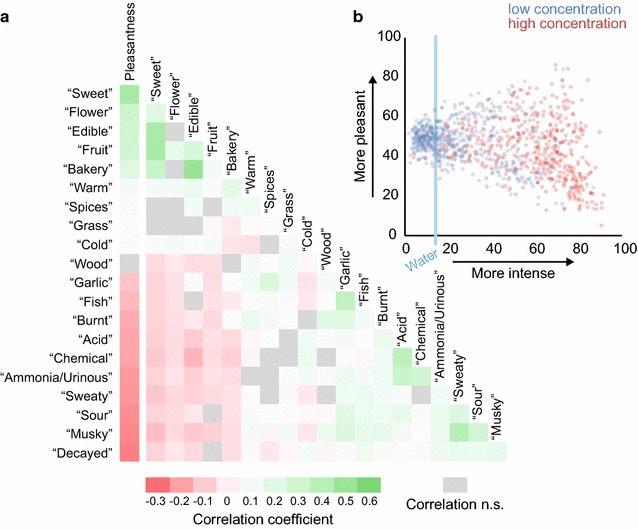
Correlations between odor quality descriptors. **a** Heat map of correlation between pleasantness ratings and the ratings of 20 descriptors. Correlations that are not statistically significant (after Bonferroni correction; N = 41,289, p > 0.000238) are indicated in *grey*. **b** Average intensity and pleasantness ratings for 1000 stimuli

The descriptors used in this study do not refer to independent qualities of molecules (Fig. 7a). Stimuli that were perceived as “fruit” were more likely to be perceived as “sweet.” The descriptors “sweet,” “flower,” “edible,” “fruit,” and “bakery” were strongly correlated with a high rating for pleasantness. In contrast, the odor quality descriptors “decayed,” “musky,” “sour,” and “sweaty” were correlated with a low pleasantness rating. Between the odor quality descriptors, the highest correlation was between “edible” and “bakery,” followed by “sweet” and “fruit,” “sweet” and “edible,” and “musky” and “sweaty.” Some descriptors were mutually exclusive and therefore negatively correlated. The strongest negative correlation was found between “edible” and “chemical,” followed by “sweet” and “musky,” and “sweet” and “sweaty.”

None of these negative correlations between descriptor pairs was as strong as the correlation between pleasantness and intensity (Fig. 7b). Very unpleasant stimuli tended to be perceived as very intense. However, as can be seen in Fig. 7b, the relationship between perceived intensity and pleasantness is more complex. Weak stimuli were perceived as neither very pleasant nor very unpleasant. Both the 29 most unpleasant and the 9 most pleasant stimuli had an intensity rating over 50.

### Subjects’ own words

**Fig. 8:**
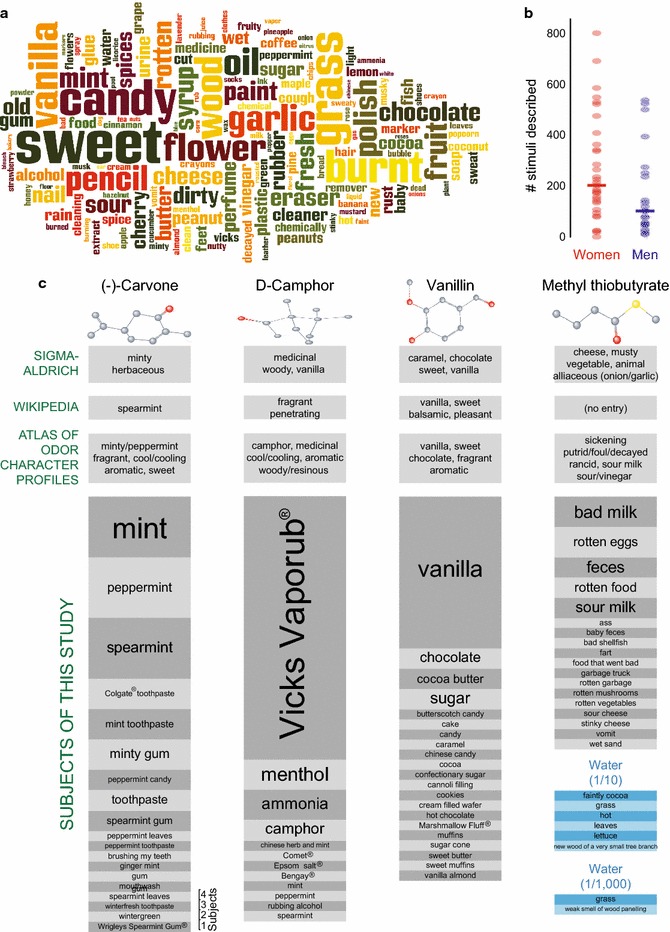
Subjects’ own words. **a** A word cloud in which font size represents the frequency with which words describing odor quality were used. **b** The number of stimuli that each of the 55 subjects described in their own words. Individual data are shown as *dots*, median as *line*. **c** Semantic odor descriptors for (−)-carvone (1/10), D-camphor (1/10), vanillin (1/10), and methyl thiobutyrate (1/1000). Published descriptors from Sigma-Aldrich Flavor and Fragrance Catalogue, Wikipedia, and the five descriptors with the highest applicability from the Dravnieks odor atlas [21] (*top*) and self-generated descriptors provided by subjects for the same 4 odor stimuli as well as water “diluted” 1/10 or 1/1000 (*bottom*). In the chemical structures, atoms are colored as follows: carbon (*gray*), oxygen (*red*), sulfur (*yellow*)

In addition to rating intensity, pleasantness, and 20 descriptors, subjects were given the opportunity to describe the stimuli in their own words (Fig. 2a; Additional file 1). Overall, the words used by the subjects were subsets of descriptors used by fragrance professionals [21]. Words such as “sweet,” “burnt,” “grass,” “candy,” and “vanilla” were common (Fig. 8a). However, there were also some idiosyncrasies. Women tended to describe more of the stimuli than men (Fig. 8b). Providing self-generated descriptors was optional, and subjects used their own words to describe between 2 and 803 of the 1000 stimuli (median: 173). The subject who provided descriptors for 2 stimuli used only two words (“burnt” and “paint”). The subject who described the most stimuli used 7160 words, 766 of them unique. The most common words used by this subject were “sweet,” “pencil,” and “eraser,” which were used 156, 139, and 133 times, respectively. Both of these subjects had above average olfactory performance (Fig. 2b), suggesting that the variability in the number of described stimuli is due to behavioral rather than perceptual variability. Subject-generated descriptions were similar to published descriptors found in the Sigma-Aldrich Flavor and Fragrance catalogue, on Wikipedia, or in the Dravnieks odor atlas (Fig. 8c). One notable difference was that subjects used product names such as Vicks VapoRub^®^, Marshmallow Fluff^®^, and Bengay^®^, to describe molecules (Fig. 8c). Few subjects attempted to describe the smell of water (Fig. 8c).

### Structure-odor relationships

**Fig. 9:**
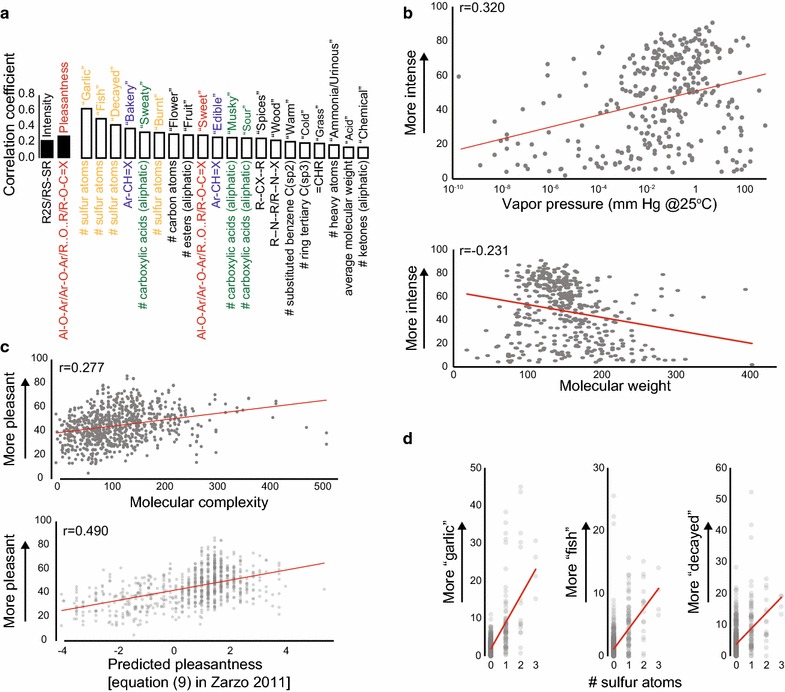
Predicting perception. **a** The strongest positive correlations between a molecular feature and intensity, pleasantness, and descriptor ratings. All correlations shown are statistically significant (after Bonferroni correction; p < 0.000235). **b** Perceived intensity and vapor pressure (*top*; limited to the 319 molecules with available vapor pressure information) and perceived intensity and molecular weight (*bottom*) (p < 1E − 05). **c** Pleasantness and molecular complexity (*top*), and pleasantness and molecular features computed using Eq. (9) in [4]. Eq. (9) equates perceived pleasantness with −2.62 + 0.23* number of atoms (excluding H) + 1.58* presence of oxygen −1.96* presence of sulfur −2.58* presence of an acid group −1.89* presence of an amine group (*bottom*) (p < 1E−14). **d** The number of sulfur atoms and ratings for “garlic” (*left*), “fish” (*middle*), and “decayed” (*right*). In all panels, only stimuli diluted at 1/1000 are included in analysis of intensity so that only stimuli diluted to the same level are compared; and only the 778 stimuli perceived to be more intense than water (14.44) were included in the analysis of pleasantness and odor descriptors

This dataset makes it possible to investigate the relationship between the physical features of molecules and their perceptual qualities. Figure 9a shows the physical features of the molecules that have the strongest positive correlation with the ratings of intensity, pleasantness, and each of the 20 descriptors. The most basic perceptual quality in any modality is the intensity with which the stimulus is perceived. 437 stimuli in this study were diluted 1/1000, and this subset of stimuli can be used to investigate which physical features predict the perceived intensity of a molecule. We found a positive correlation between the vapor pressure of a molecule and its intensity (Fig. 9b, top). The molecular feature that had the strongest positive correlation with perceived intensity of the stimuli diluted 1/1000 in this study is the presence of an atom-centered fragment that contains a sulfur atom (Fig. 9a). An atom-centered fragment consists of a central atom that is surrounded by one or several shells of atoms that are separated from the central one by the same topological distance. We also replicated the previously reported negative correlation between molecular weight and perceived intensity (Fig. 9b, bottom) [2].

Another perceptual quality of odorants that has been predicted using molecular features is pleasantness [6]. The proposal that molecules with higher molecular complexity are more pleasant [5] was replicated by our dataset (Fig. 9c, top). Another prediction of perceived pleasantness was based on the observation that the number of atoms (excluding H) and the presence of an oxygen atom results in molecules that are perceived to be more pleasant, whereas the presence of sulfur, an acid group, or an amine group make it less pleasant [4]. This prediction was also replicated by our dataset (Fig. 9c, bottom).

The goal of most research that attempts to link perceptual and molecular determinants of a smell is to predict whether certain semantic descriptors are likely to be applied to the smell of a molecule. Our data show that this differs between odor quality descriptors (Fig. 9a). For “chemical” and “acid” there were only weak correlations with chemical features. The strongest correlations were seen between the odor quality descriptors “garlic,” “fish,” and “decayed” and the number of sulfur atoms in the molecule (Fig. 9d).

## Discussion

We present a dataset that captures the sensory perception of 480 different molecules at two different concentrations as experienced by 55 demographically diverse healthy human subjects. Subjects rated intensity, pleasantness, familiarity, and applied 20 odor descriptors. 40 stimuli (20 molecules at two concentrations) were presented to the subjects twice. Subjects were capable of matching intensity ratings to the concentration of the stimulus molecules: 98 % of the molecules that subjects perceived to be stronger than water (intensity rating of 14.44) were perceived to be more intense at the high concentration than at the low concentration (median intensity rating difference: 33.2).

We discovered a strong influence of familiarity on the semantic description of odorants. In addition, the odor quality descriptors applied to the stimuli replicated how descriptors were applied by experts in many cases [21]. Among the molecules used both in this study and in Dravnieks’s study, diethyl disulfide, was the most representative of the descriptor “garlic” in both studies. Similarly, the molecules most representative of “flower” (2-phenylethanol), “decayed” (methyl thiobutyrate), “sweaty” (isovaleric acid), and “spicy” (eugenol), were the same in the two studies.

However, we also found marked differences in how descriptors were used by our untrained subjects and experts. For example, subjects used “musky” to describe unpleasant body odors. In contrast, experts use “musky” to describe compounds naturally sourced from animal glands or their synthetic analogues. These are often used as base notes in perfumery, and experts associate musks with pleasant descriptors such as “sweet,” “powdery,” and “creamy.” However for our subjects, “musky” had a negative correlation with pleasantness, and was instead correlated with the descriptor “sweaty.” The molecule rated as most “musky” in this study was isovaleric acid, which experts do not rate as “musky” [21]. The five molecules that Dravnieks lists as representative of the “musk” descriptor are also rated “fragrant” and “perfumery” by experts [21]. Therefore, the word “musky” has a colloquial meaning that is different from its technical meaning in perfumery.

### A new dataset for human psychophysics research

This dataset differs from most other sources of information about how different molecules are perceived. First, we included molecules that are usually considered to be odorless, like water, glycerol, and citric acid, to obtain data on an outgroup missing from human olfactory research. We also included molecules with unfamiliar smells not easily associated with descriptors commonly used to classify odorants. As a consequence, subjects did not recognize more than 69 % of the stimuli. Most existing datasets consist largely of molecules that are representative of specific descriptors. This practice leads to the danger that language conventions used to describe odorants and not odor perception itself are studied. Avery Gilbert and Mark Greenberg succinctly summarized the dangers of this approach when they wrote that “we are creating a science of olfaction based on cinnamon and coffee” [24] (page 329). Including odorless molecules and those that do not match common semantic descriptors allows for a more comprehensive analysis of human smell perception.

A second feature of our dataset is that it includes familiarity ratings. In datasets produced by Dravnieks [21] or Arctander [23], industry experts evaluated familiar molecules of commercial importance. By studying the perception of unfamiliar odorants, we avoid the pitfall of subjects conflating odor quality with past odor associations.

A third feature that distinguishes this dataset from many other sources of information about olfactory perceptual qualities is that we not only report a population average, but also how individual subjects rated the stimuli. This information is useful because olfactory perception depends not only on the molecular features of the stimulus, but also on the perceptual system of the perceiving subject [9, 12–16].

To stimulate further analysis of the data in this study, we are making the entire dataset freely available (Additional file 1). Our analysis here is primarily concerned with predicting how different molecules are perceived. However, the dataset enables the investigation of other topics, for example differences in perception between different demographic groups [19]. Perceptual correlations between stimuli (Fig. 5b) can be used to arrange molecules in a perceptual odor space, or to investigate the underlying mechanisms of shared perception.

### Predicting intensity

There are two complications to predicting perceived intensity. First, the intensity of a given molecule at a given dilution is not only dependent on the interaction between the molecule and the perceptual system, but also on how many of the molecules will reach the odorant receptors. How many molecules will be released from the dilution depends on vapor pressure, solubility in the solvent, and operational factors during the assay such as room temperature, humidity, etc. The vapor pressures cited in Fig. 9b pertain to pure molecules and do not take into account interactions with the solvent. Once the molecules arrive at the olfactory epithelium, the efficiency with which they reach the hydrophobic binding pockets of odorant receptors depends on their lipophilicity. Consequently, perceived intensity has a positive correlation with vapor pressure (r = 0.320; p = 4.99E−09) and a negative correlation with a measure of hydrophilicity (squared Moriguchi octanol–water partition coefficient (logP^2^); r = −0.321; p = 4.45E−09).

The second complication is that different molecules have different concentration–response functions, which determine the relationship between the dilution of the molecule and its perceived intensity [25]. Because the relation between dilution and perceived intensity differs between different molecules, it is possible that a molecule perceived to be stronger than another molecule at one dilution will be perceived as weaker than the other molecule at a different dilution. This is clearly illustrated by the two molecules highlighted in Fig. 4b. While methyl salicylate was perceived to be more intense than methyl caprylate at the 1/1000 dilution, methyl caprylate was perceived to be more intense than methyl salicylate at the 1/100,000 dilution. Because of this complication, it is not enough to predict the perceived intensity of a molecule at a given dilution. Instead, the parameters of the concentration–response function have to be predicted. The two dilutions used for the molecules in this study are not sufficient to determine the parameters of the concentration–response functions, but they allow for more sophisticated prediction of perceived intensity than those based only on a single dilution or detection threshold.

The 437 molecules that were presented in this study at a dilution of 1/1000 were perceived to be of very different intensities. 63 of the 437 molecules were perceived to have lower intensity than water (intensity rating 14.44). If the ratings of all odorless molecules are drawn for the same distribution of perceptual noise, this implies that around 126 of the molecules used are undetectable at a 1/1000 dilution. At the other extreme, the 1/1000 dilution of methyl thiobutyrate and other molecules were perceived to be very strong stimuli. The previously reported correlation between vapor pressure and perceived intensity as well as the negative correlation between molecular weight and perceived intensity were reproduced in this dataset [2]. The data are clearly more complex than correlations between single molecular features and perceived intensity can capture. For example, low perceived intensity was reported with molecules of very low and very high vapor pressure. We also discovered that a single molecular feature, the presence of a certain sulfur-containing atom-centered fragment, has a positive correlation to perceived intensity that is almost as strong as the correlation between molecular weight and intensity.

### Predicting pleasantness

In this work we replicated the finding that perceived pleasantness correlates with molecular complexity [5]. We also confirm the observations that perceived pleasantness correlates with the number of atoms (excluding H), that the presence of an oxygen atom results in molecules that are perceived to be more pleasant, whereas the presence of sulfur, an acid group, or an amine group make it less pleasant [4]. A third prediction for pleasantness [6] that made it possible to predict pleasantness with r~0.5 was partially based on molecular features that were not provided by the version of the cheminformatic software we used (Dragon 6) [26, 27]. However, the chemical features from the model in [6] provided by our version of the software, showed the same relationship with pleasantness in our data set.

The presence of a certain atom-centered fragment containing an oxygen atom had the strongest correlation with pleasantness. Three previous models that predict pleasantness performed well on our independent dataset, suggesting that a combination of these models might outperform any single model.

### Predicting odor qualities

Most research into structure-odor relationships is concerned with explaining why a given semantic descriptor, such as “musky” or “camphorous” is commonly applied to some molecules, but not to others [1, 28, 29]. Among the 20 descriptors used here, the strongest correlation between a descriptor and a molecular feature was between the semantic descriptor “garlic” and the number of sulfur atoms in the molecule (r = 0.63), while the strongest positive correlation of the descriptor “chemical” with any molecular feature was 0.14. The differences in the strength of correlations between semantic descriptors and molecular features suggest that the application of some semantic descriptors (“garlic” or “fish”) can easily be predicted, whereas the application of others (“chemical” or “acid”) is either impossible or much more complex to predict.

A plausible explanation for this observation is that all semantic descriptors that are assigned to odorants must be learned by association. It may be that the situations in which subjects formed an association between the word “garlic” and a specific smell are very similar between subjects. The associations between the word “chemical” and a specific smell on the other hand are probably different between subjects. All molecules are chemicals and the descriptor “chemical” was often used by subjects to describe a wide variety of molecules when they could not identify the smell. This suggests that some descriptors have a single reference odorant, with which they are strongly associated whereas other descriptors have several or no reference odorants. This results in weak associations between the descriptor and the reference molecules that vary between individuals.

## Conclusions

Psychophysical data with a larger number of chemically diverse molecules will increase our understanding of the relationship between stimulus and perception in olfaction. The data presented here can be used to guide experimental design for a variety of olfactory neuroscience research projects such as fMRI studies, or the study of perceptual phenomena like adaptation or mixture interactions. The data can be used, for example, to choose stimuli whose perception is variable or stereotyped, or perceptually similar or dissimilar.

The data presented here reveal that the assignment of descriptors depends strongly on familiarity with the smell. We presume that subjects only applied a given descriptor to a stimulus when they could retrieve the memory of the reference smell that they associated with the descriptor. The specificity of the reference smell depends on the odor quality descriptors. “Spices” is a semantic descriptor that can trigger a variety of different smell associations, whereas “garlic” refers to a more specific type of smell. We anticipate that only descriptors with an unambiguous reference odorant can be predicted based on molecular features.

Another problem with verbal descriptors is that they are culturally biased. The current standard set of 146 Dravnieks descriptors was developed in the United States in the mid-1980s and is increasingly semantically and culturally obsolete. Even if these descriptors were updated to be current and relevant across different nationalities and cultures, it is unlikely that semantic descriptors will ever cover the entire olfactory perceptual space. Moreover, because the existing descriptors were developed with a small list of stimuli, new untested molecules or complex mixtures of molecules may lack appropriate semantic descriptors. To circumvent the limitations of verbal descriptors, an alternative semantic-free approach to predict similarity between stimuli based on molecular features [30] should be pursued. Initial implementations of this method have shown astonishing success, producing a correlation of r = 0.85 between predicted and empirically-determined stimulus similarity [30]. Predicting perceptual similarity between olfactory stimuli would result in a complete and comprehensive ability to predict the perception of any molecule. Once robust methods for predicting perceptual similarity have been developed, it will be possible to predict verbal descriptors for a new molecule by predicting its similarity to a signature odorant representative of a given descriptor.

Studies that aim to predict smell perception based on molecular features have been given a boost by the introduction of Dragon software, which calculates thousands of different molecular features [26, 27]. This large collection of molecular features frees researchers from guessing what features of molecules influence how they are perceived, and makes it possible to test a wide variety of molecular features to find those that play a role in determining a molecule’s smell. However, the large number of molecular features available for building formal models of structure-odor relationships also brings the danger of overfitting. An overfitted model fits the data that was used to create it well, but it has poor predictive performance. Overfitting often occurs when a model has too many parameters relative to the number of observations. Reducing the likelihood of overfitting by increasing the number of molecules that can be used to test formal models was a major motivation behind generating this dataset.

Importantly, we have used a subset of the data presented here for a competitive modelling challenge in collaboration with IBM Research, Sage Bionetworks, and DREAM challenges [31]. For this competition, predictive models were built based on a training set containing a subset of the stimuli and then evaluated using a different subset of the stimuli. The DREAM Olfaction Prediction Challenge aims to develop the most comprehensive computational approach to date to predict olfactory perception based on the physical features of the stimuli. The results of this challenge will be published elsewhere.

## Methods

### Subjects

Healthy subjects between the ages of 18 and 50 were recruited from the New York City metropolitan area and tested between February 2013 and July 2014. 61 subjects completed all 10 study visits. The remaining subjects dropped out before all 10 visits were completed, or were not invited back after the first visit at our discretion. We quantified olfactory performance by measuring the correlation between intensity ratings of 40 stimuli that were presented twice (Fig. 3a, top and Fig. 2b, blue symbols), and the number of molecules that subjects rated as more intense at the high versus low concentration (Figs. 2b, red symbols and 4b). The six subjects with the lowest average rank of both measures were excluded (Fig. 2b, black symbols), leaving 55 subjects (33 female) whose data comprise the results of this paper. Of these, 25 self-identified as black, 17 as white, 5 as Asian, and 2 as Native American. 12 self-identified as Hispanic (Additional file 1). The median age of the subjects was 35 (Fig. 2c).

### General psychophysics procedures

The subjects were tested in the Rockefeller University Hospital Outpatient Clinic. Psychophysical tests were self-administered and computerized using custom-written software applications that ran on netbooks. To prevent errors, all odorant vials used in this study were barcoded. Subjects scanned each odorant vial containing the stimulus before opening the vial, and were only prompted to proceed if the correct vial was scanned.

Subjects opened the vial, sniffed the contents, and were asked if they could smell anything. If the answer was “no,” they were directed to move on to the next stimulus. If the answer was “yes,” they were asked if they know what the smell is. If they answered “yes,” they were given a chance to describe the smell (Fig. 8). Then they were asked a series of 23 questions about the smell (Fig. 2a). For each question, they were presented with a slider that could be moved along a line. The final position of the slider was then translated into a scale from 0 to 100. The first three questions asked how familiar, strong, and pleasant the smell was. For these three questions, the slider started in the middle of the line (position 50) and subjects were required to move it. The other 20 questions were how well each of 20 descriptors (“edible,” “bakery,” “sweet,” “fruit, “fish,” “garlic,” “spices,” “cold,” “sour,” “burnt,” “acid,” “warm,” “musky,” “sweaty,” “ammonia/urinous,” “decayed,” “wood,” “grass,” “flower,” and “chemical”) applied to the smell. For these questions, the slider started at the bottom of the line (position 0) and subjects were not required to move it. The 20 descriptors were chosen because they are broad enough to be applied to enough stimuli in our set to allow for the development of models that predict the application of the descriptor based on molecular features. Other descriptors such as “pineapple,” “cork,” and “wet paper” are so specific that they are applied to relatively few molecules [21].

Each subject came to the Rockefeller University Outpatient Clinic for ten visits, and profiled 100 stimuli during each visit. The order of stimuli was randomized differently for each subject. Although there are likely sequence effects for individual ratings, such as a moderately pleasant odorant being rated as more pleasant when it follows a series of very unpleasant odorants than when it follows a series of several very pleasant odorants, these are averaged out in the pooled data. Subjects carried out the study at their own pace with a typical pace of 1 stimulus/min.

### Stimuli

Stimuli were presented in vials as 1 mL of the diluted molecule in paraffin oil. Information about the stimuli and their dilutions can be found in Additional file 1. The chemicals were >97 % pure with a median purity of 98 %. This is a limitation of this dataset because 3 % impurity can have an impact on the percept, especially when the molecule itself is odorless, but the impurity has a smell.

### Molecular features

Molecular complexity (Figs. 1b, 9c, top) was computed using the Bertz/Hendrickson/Ihlenfeldt formula [7]. It is a rough estimate of the complexity of a molecule, and considers the variety of elements in the molecule as well as structural features including symmetry. Stereochemistry is not used as a criterion. In general, large compounds are more complex than small compounds. The correlation between molecular complexity and number of atoms (excluding H) among the 480 molecules used here is 0.88 (p = 1.2E−156), but high symmetry and the lack of diversity in atom types results in lower complexity. The complexity values were obtained from NCBI PubChem. Vapor pressures (Fig. 9b, top), which were experimentally measured or calculated by others, were obtained from online databases such as those at NCBI PubChem, The Goods Scents Company, Givaudan, and Sigma-Aldrich.

Molecular features (Fig. 9a) were calculated using Dragon 6 software (Talete) [26, 27]. Of the 4885 molecular features, only the following categories were included in the analysis presented here: atom-centered fragments (115 descriptors), constitutional indices (43 descriptors), functional group counts (154 functional descriptors), molecular properties (20 descriptors), and ring descriptors (32 descriptors). Topological indices, walk and path counts, connectivity indices, information indices, 2D matrix-based descriptors, 2D autocorrelations, Burden eigenvalues, P_VSA-like descriptors, ETA indices, edge adjacency indices, geometrical descriptors, 3D matrix-based descriptors, 3D autocorrelations, RDF descriptors, 3D-MoRSE descriptors, WHIM descriptors, GETAWAY descriptors, Randic molecular profiles, atom-type E-state indices, CATS 2D, 2D atom Pairs, 3D atom Pairs, charge descriptors, and drug-like indices were not included in the analysis. Molecular features that had the same value for more than 98 % of the molecules used here were also excluded from the analysis.

### Word cloud

The word cloud in Fig. 8a shows how frequently certain words were used by the subjects to describe the smells of the stimuli. It was produced with the Wordle program at http://www.wordle.net and represents the frequency of word usage by font size. The program was set to remove common English words (“and,” “but,” “or,” etc.), and the following words were manually excluded because they did not describe perceptual qualities: “smell,” “smells,” “smelly,” “smelling,” “odor,” “sort,” “also,” “something,” “kind,” “mixed,” “maybe,” “flavor,” “flavored,” “strong,” “slightly,” “mildly,” “type,” “background,” “like,” “used,” “hint,” “mild,” “bit,” “reminds,” “mix,” “scented,” “faintly,” and “scent.”

## Additional file

10.1186/s12868-016-0287-2 Study data. This spreadsheet lists the odorants used in this study (source, name, CID, C.A.S. number, and dilutions), subject demographic information (gender, race, ethnicity, and age), and the psychophysical dataset used for all the analyses in this paper.
